# On doctors’ accountability and flight deck safety

**DOI:** 10.3325/cmj.2015.56.385

**Published:** 2015-08

**Authors:** Alpo Vuorio, Tanja Laukkala, Navathe Pooshan, Bruce Budowle, Anne Eyre, Antti Sajantila

**Affiliations:** 1 Mehiläinen Airport Health Centre, Vantaa, Finland *alpo.vuorio@gmail.com*; 2 Mehiläinen, Kielotie, Vantaa, Finland; 3 The Maitland Hospital, Maitland, Australia; 4 Institute of Applied Genetics, Department of Molecular and Medical Genetics, University of North Texas Health Science Center, Fort Worth, Texas, USA and Center of Excellence in Genomic Medicine Research (CEGMR), King Abdulaziz University, Jeddah, Saudi Arabia; 5 Trauma Training, Coventry, UK; 6 University of Helsinki, Department of Forensic Medicine, Helsinki, Finland

People have an inherent tendency to view events as being more predictable than they may actually be, especially in hindsight. This predisposition is known as hindsight bias. However, putting hindsight bias aside, one should consider the discussion in the media raised by the recent Germanwings Flight 9525 air accident from a medical point of view. The probability that aircrafts are used to assist suicide is exceedingly low. We have recently estimated that suicide accounts for about 0.3% of fatal accident cases in general aviation, which would be approximately 8 cases in the USA between 2003-2012 (1); but this figure is likely to be even lower in commercial air transport. However, in commercial air transport the consequences are severe, in that one accident can cause many deaths.

The recent accident has already prompted improvements in aviation safety management. Operational regulations have been changed worldwide, and now include, as a few countries already practice, that whenever either the pilot or co-pilot steps out of the cockpit, a second member of the crew, such as a flight attendant, is required to enter. However, as a measure of preventive security, this practice occurs at the last moment to deter a possible tragedy such as experienced with Germanwings Flight 9525. While we support the new regulations, we also call for active prevention at a much earlier stage in the safety management process. Necessary and open discussion of such events is confounded by the fear that massive and inappropriate media coverage of a celebrity suicide could instigate a copycat effect (2), and therefore preparedness is essential.

## Previous aircraft assisted suicides in commercial air transport

In 1982, Japan Airlines Flight 350 plunged into Tokyo Bay (3). Although the co-pilot and the flight engineer were in the cockpit, they were not able to completely overcome the captain’s attempt to crash the plane. The captain survived and was shown to have paranoid schizophrenia, and was found not guilty by reason of having acted under delusions. The Japanese government investigators attributed the incident partly to a poor medical examination system of the airline and an airline physician’s erroneous decision to allow the pilot to return to duty. Among other issues, this accident indicates the seriousness of the challenge of preventing aircraft assisted suicides.

In November 2013, LAM Mozambique Airlines Flight 470 crashed in Namibia (4). In an unnervingly similar fashion to the Germanwings flight, the co-pilot had left the cockpit, and the pilot changed the altitude setting to below ground level and manually switched the settings to maximum speed. The co-pilot was heard pounding on the outside of the cockpit door from the cockpit recorder. Devastating as this accident was, it did not lead to demands for two people to be in the cockpit at all times.

## Lessons learned from Germanwings aircraft-assisted suicide

Significant suicide related issues arise from the deliberate crashing of an aircraft carrying many passengers. Such cases do not classify as mere suicides since the pilot must realize that as the aircraft shatters, a large number of people will also be killed. Therefore, such events alternatively can be classified as extended suicides or even as homicide-suicides (5,6), although the literature on murder-suicides suggests that such events more commonly involve members of the family or close acquaintances, and those involving strangers are rare (7). For example, in 2001 in Switzerland the homicide-suicide offending rate per 100 000 was 0.10 and victimization rate 0.25 (8). This translated into a rate of 2.95 per 1 000 000 eligible households for all murder-suicides.

Medical professionals will never be able to prevent all aircraft assisted suicides or murder-suicides. All suicides are not directly related to medical factors (9). But to reduce the chances, those doctors involved in aircraft safety investigations will need better training in detecting tell-tale signs that indicate risk of serious suicidal ideation in pilots. Also, additional scrutiny should be implemented during the process of conferring pilots’ medical certificates. The issues facing air safety may have implications beyond aviation specialists to other medical experts. In 2014 the Finnish Safety Investigation Authority recommended that additional training, especially of psychiatrists, neurologists, and cardiologists, also should include the option of consulting the pilot’s medical examiner or the Transport Safety Agency (10). In fact, this option is relevant to any doctor. Specialists should be actively consulted to help set minimum requirements of pilot health issues to facilitate screening. The new philosophy in safety investigations is that while it is important to study accidents in detail, good practices and a study of how pilots and aero-medical doctors are able to provide required performance under expected and unexpected conditions also are necessary (11). One good practice is to set a culture that promotes voluntary reporting regarding cabin crew health. Such a reporting system could be developed to identify and provide useful confidential data for aviation health care workers.

Initially, the focus on doctors’ responsibility and accountability should be on supporting those who have lost loved ones, safety workers, local inhabitants in the area of the accident, and all those colleagues involved in addressing this tragic set of circumstances and its aftermath. Future improvements should be well thought out with consideration from a holistic perspective of this emotive and difficult issue. With the experience gained from the Germanwings accident and several others, changes are already being made for safety and security. We strongly urge the same commitment be made to determining the signals of pilot health risk as both avenues will reduce the risk of another pilot suicide attempt.
